# Variability and Advancements in ChatGPT Risk of Bias Assessments: A Replication and Comparative Analysis

**DOI:** 10.1111/jebm.70046

**Published:** 2025-06-17

**Authors:** Jules Descamps, Matthieu Resche‐Rigon, Guillaume Draznieks, Cesar Quirino, Rémy Nizard, Pierre‐Alban Bouché

**Affiliations:** ^1^ Université Paris Cité Paris France; ^2^ Service de Biostatistique et Information Médicale (SBIM) UMR 1153 CRESS Inserm, Hôpital Saint‐Louis, APHP Paris France; ^3^ Département Chirurgie Orthopédique Hopital Lariboisière, AP‐HP Paris France; ^4^ Ecole Polytechnique Palaiseau France; ^5^ DeepDocs AI LLC Phoenix Arizona USA

**Keywords:** data extraction, large language models (LLMs), risk of bias

1

Dear Editor,

We read with great interest the letter by Kuitunen et al., in which they evaluate the performance of ChatGPT‐4o in conducting risk of bias (RoB) assessments using the Cochrane RoB2 tool. Their study sampled 100 randomized controlled trials (RCTs) from recent meta‐analyses published in top‐tier medical journals, prompting ChatGPT‐4o to provide an overall rating (“low,” “some concerns,” or “high”) and domain‐specific ratings for each study. The authors found that the interrater agreement was generally slight to poor, aligning with previous smaller scale observations and highlighting that ChatGPT‐4o's default outputs may be overly optimistic when determining bias levels.

We commend the authors for their systematic approach. Their standardized prompt and focus on RoB2 across a larger sample of RCTs strengthen the validity of their findings. Furthermore, their interrater reliability analyses revealed low correlation coefficients, which underscores the challenges inherent in automating such nuanced evaluations. These results add valuable quantitative data to an area where robust evidence is still emerging.

Nevertheless, we wish to highlight certain methodological limitations that warrant further consideration. First, the inclusion of five duplicate articles [1, 2, 3, 4, 5]—each cited in two different meta‐analyses—introduced a situation in which identical articles had identical “ground truth” RoB2 assessments yet received different evaluations by ChatGPT‐4o, illustrating variability in large language model (LLM) responses. For instance, for this article [4], D5 was either low and some concerns. Second, the rule‐based determination of the overall RoB (low/some concerns/high) from domain‐specific ratings itself is algorithmic and does not necessarily require a generative LLM for completion, suggesting that a simpler automated “classification” method might suffice for this aspect. Third, as Kuitunen et al. acknowledge, relying on a single LLM extraction to generate these assessments may be inherently limited, particularly if the model's output can shift based on small prompt changes or session variability.

To address these issues, we replicated the methodology in the same data set of 100 RCTs using both the original ChatGPT‐4o and its updated iteration, 4o‐new. Additionally, we employed a sophisticated framework (4o‐fram) that integrates 4o as an input for processing .jsonl files associated with full‐text articles. The 4o‐fram framework (DAM Assess Version 1.25.01; DeepDocs LLC) utilizes a systematic multi‐step approach; it applies a predefined evaluation grid to OCR‐converted full‐text PDFs using a language model, then structures the results into a clean, analyzable Excel table (Figure S1). We also tested OpenAI's newer “o1” model using exactly the same prompts. We replicated the analysis using our own dataset and models, comparing the original ChatGPT‐4o results reported by Kuitunen et al. to new outputs from the same model (4o‐new) and the updated OpenAI model (o1). Although we did not compute weighted Fleiss’ kappa due to reproducibility challenges, our plain Fleiss’ kappa and proportion agreement tables provide a direct comparison of performance across different models and domains. The variability between the original 4o and our 4o‐new outputs likely reflects intrinsic fluctuations in LLM responses (Figure 1). For instance, in Domain 1 (D1), the original ChatGPT‐4o showed moderate agreement with a Fleiss’ kappa of 0.31 (95% CI 0.25–0.36), whereas our new iteration (4o‐new) dropped to −0.05 (95% CI −0.16 to 0.05). The 4o‐fram framework, which utilizes ChatGPT‐4o with .jsonl files linked to full‐text articles, showed notable improvements in agreement across most domains. For instance, in D1, it achieved a Fleiss’ kappa of 0.37 (95% CI 0.29–0.46), substantially higher than the 4o‐new iteration and the original 4o outputs. This demonstrates the potential of leveraging full‐text data and structured input frameworks to enhance LLM performance in systematic assessments. In contrast, the updated o1 model improved agreement with a kappa of 0.11 (95% CI 0.03–0.19) (Table 1).

**FIGURE 1:**
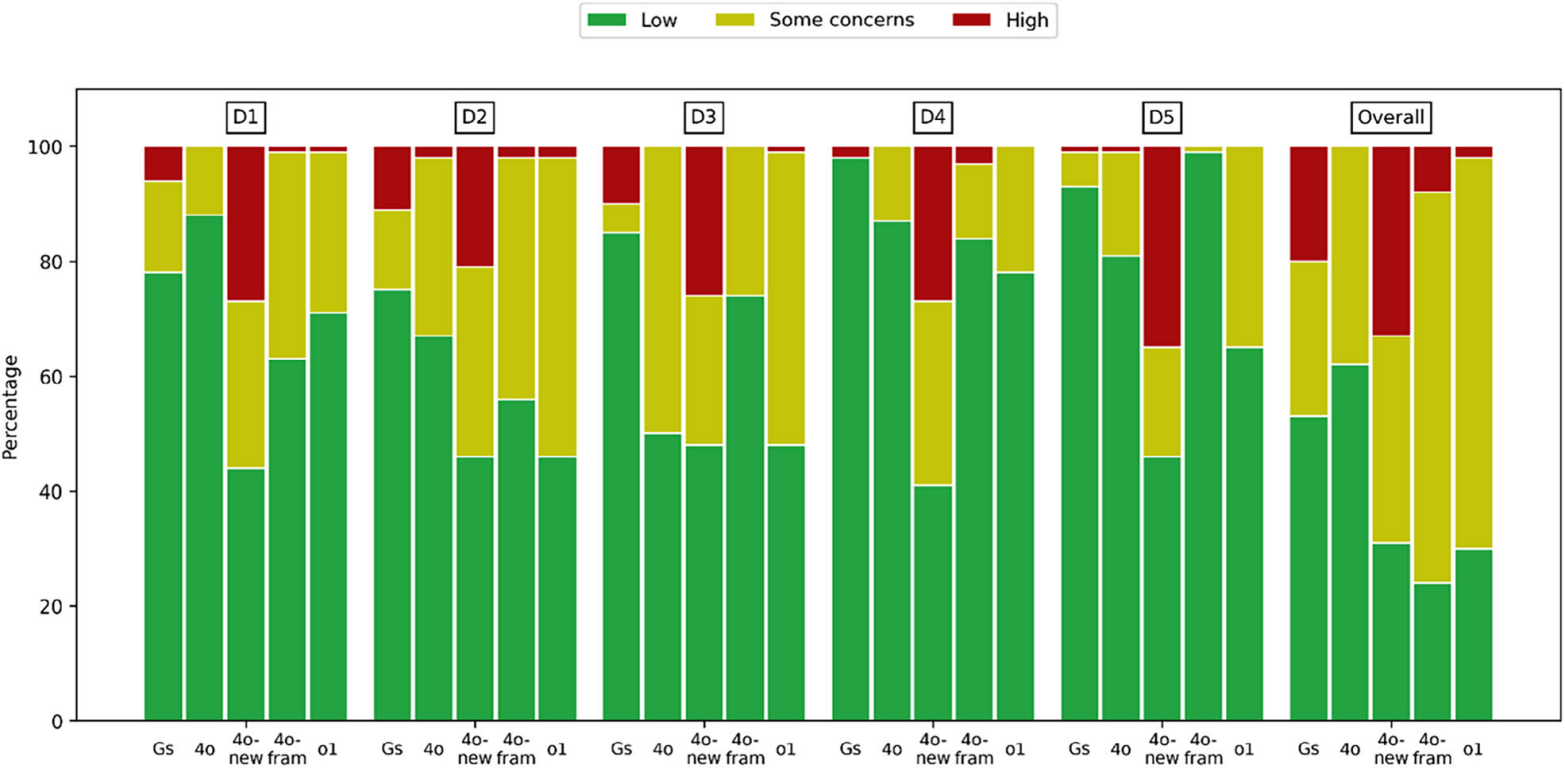
Comparison of the categorization of risk of bias assessments in the five domains and overall between Gs: extracted assessments; 4o: ChatGPT‐4o from Kuitunen et al.; 4o‐new: ChatGPT‐4o from Descamps et al.; 4o‐fram: ChatGPT‐4o with DeepDocs framework; o1: ChatGPT‐o1; D1: bias arising from the randomization process; D2: bias due to deviations from the intended interventions; D3: bias due to missing outcome data; D4: bias in the measurement of the outcome; D5: bias in selection of the reported results.

**TABLE 1:** Fleiss’ Kappa with 95% confidence intervals (CI) across domains and raters.

Domain	4o	4o‐new	4o‐fram	o1
D1	0.31 (0.25–0.36)	−0.05 (−0.16 to 0.05)	0.37 (95% CI 0.29–0.46)	0.11 (0.03–0.19)
D2	0.04 (−0.03 to 0.13)	−0.12 (−0.22 to −0.01)	0.13 (95% CI 0.04–0.23)	−0.05 (−0.15 to 0.04)
D3	−0.07 (−0.16 to 0.01)	−0.17 (−0.27 to −0.07)	0.08 (95% CI 0.01–0.15)	−0.13 (−0.22 to −0.04)
D4	−0.07 (−0.09 to −0.04)	−0.29 (−0.38 to −0.20)	−0.07 (95% CI −0.11 to −0.04)	−0.12 (−0.16 to −0.08)
D5	−0.13 (−0.18 to −0.09)	−0.21 (−0.30 to −0.12)	−0.03 (95% CI −0.05 to −0.02)	−0.13 (−0.20 to −0.07)
Overall	0.18 (0.07–0.29)	−0.07 (−0.19 to 0.05)	0.01 (95% CI −0.1 to 0.12)	−0.16 (−0.28 to −0.05)

The proportion agreement varied widely across models and domains. For example, although the original ChatGPT‐4o achieved high agreement in certain domains (e.g., 80% in D1 and 85% in D4), the 4o‐new iteration showed lower agreement values (42% in D1 and 39% in D4). However, the 4o‐fram framework outperformed both, achieving agreement rates of 73% in D1 and 82% in D4, showcasing its robustness when using structured .jsonl inputs and full‐text data. Notably, 4o‐fram demonstrated consistently superior results compared to o1, which showed variable agreement rates (e.g., 65% in D1 and 76% in D4) (Table 2).

**TABLE 2:** : Proportion of agreement across domains and raters

Domain	4o	4o‐new	4o‐fram	o1
D1	80%	42%	73%	65%
D2	58%	38%	58%	45%
D3	50%	41%	68%	46%
D4	85%	39%	82%	76%
D5	74%	43%	92%	62%
Overall	55%	30%	40%	31%
Global	67%	38.83%	68.83%	54.17%

These differences highlight both the sensitivity of model outputs to inherent variability and the potential for improvement with newer models, but even more so through better‐designed frameworks like 4o‐fram. We also would like to balance the results, in a recent review from BMJ, RoB judgments of RCTs included in more than one Cochrane Review differed substantially. The proportion agreement from humans ranged from 57% to 81% [6], which could moderate the conclusion.

Several limitations should be acknowledged in our study. First, we did not compute weighted Fleiss’ kappa statistics due to reproducibility challenges, which may limit direct comparability with the original study's metrics. Second, our analysis was constrained to the same 100 RCTs used in the original study, potentially limiting the generalizability of our findings to broader systematic review contexts. Third, the inherent variability observed between different model iterations (4o vs. 4o‐new) highlights the challenge of reproducibility in LLM‐based assessments, which remains a significant concern for systematic implementation. Fourth, although the 4o‐fram framework showed promising improvements, it requires access to full‐text articles and specialized processing infrastructure. Finally, our evaluation focused primarily on agreement metrics rather than exploring the underlying reasons for disagreements, which could provide valuable insights for future framework development.

In conclusion, we thank the authors for contributing valuable data on the performance of ChatGPT‐4o in RoB assessments. Their findings—and our own subsequent analyses—reveal that while LLM‐assisted RoB evaluation continues to face significant limitations [7], the development of structured frameworks can significantly enhance reliability and precision. We believe that further comparative studies, alongside improvements in both model architectures and protocols (e.g., systematic prompts, consensus approaches, and advanced frameworks), will be essential to determining how LLMs can be most effectively and responsibly deployed in systematic reviews and meta‐analyses.

## Conflicts of Interest

The authors declare no conflicts of interest.

## Supporting information




**Supplementary Figure 1**. 4o‐fram (DAM Assess pipeline): From Full‐Text PDFs to Structured Excel Evaluation.
**Supplementary Table 1** Proportion of agreement across domains and raters
