# Supplementary material for: Variability and Advancements in ChatGPT Risk of Bias Assessments: A Replication and Comparative Analysis
Source: J Evid Based Med. 2025 Jun 17;18(2):e70046. doi: 10.1111/jebm.70046 (PMC12174634; doi:10.1111/jebm.70046)
Supplement: Supplementary file 1 — Supplementary Figure 1. 4o‐fram (DAM Assess pipeline): From Full‐Text PDFs to Structured Excel Evaluation. Supplementary Table 1 Proportion of agreement across domains and raters [file JEBM-18-0-s001.docx]

Supplementary materials


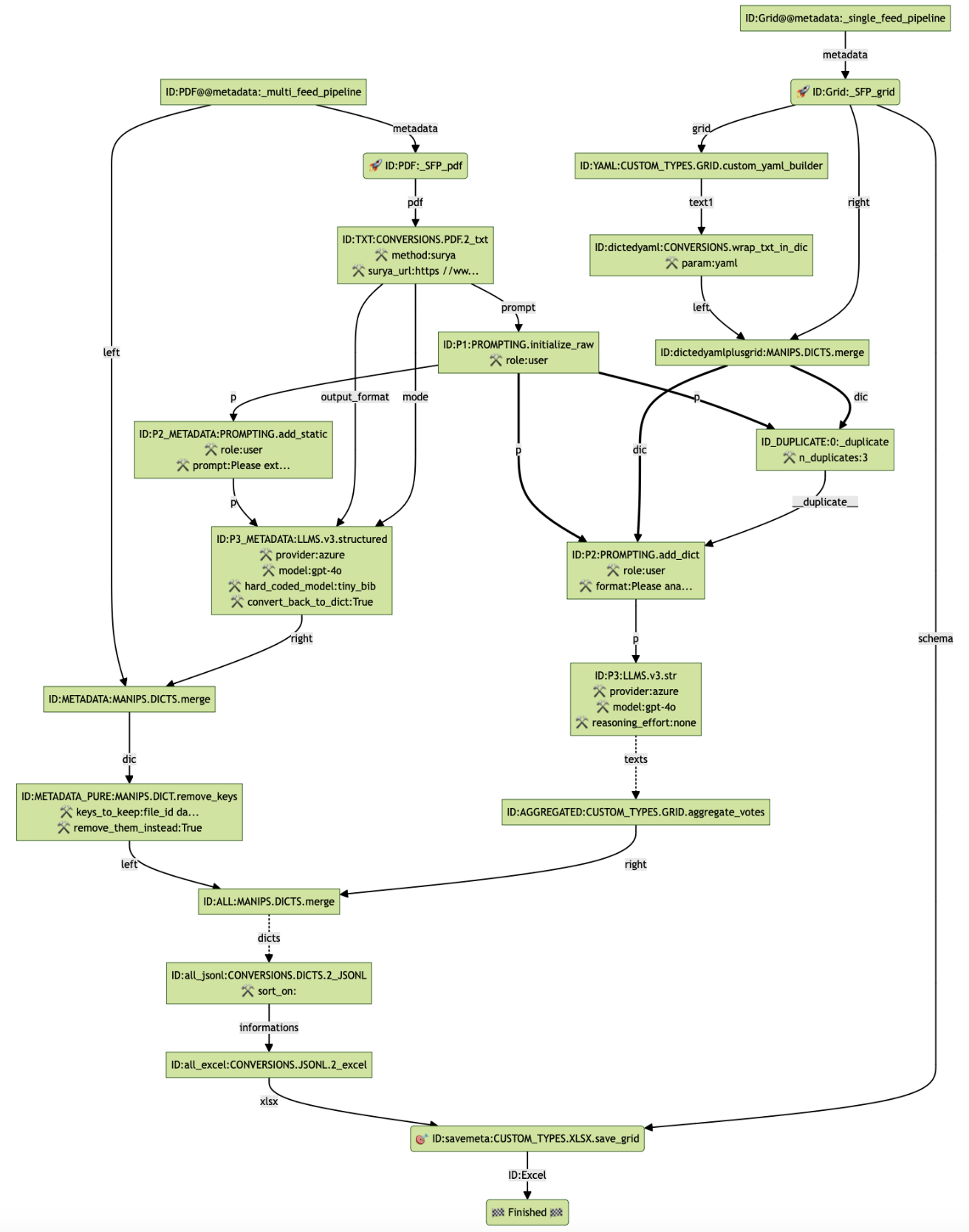


Supplementary Figure 1. 4o-fram (DAM Assess pipeline): From Full-Text PDFs to Structured Excel Evaluation

-4o-fram starts by taking full-text PDFs, which are first converted into readable text (markdown) using an AI-OCR method.

-At the same time, an assessment grid in JSON format is created from a YAML configuration file.

-Each criterion from the grid is automatically applied to the full text using prompts generated for a language model (GPT-4o) to evaluate each variable.

The model outputs are then structured and cleaned into consistent data dictionaries.

Finally, all results are merged and exported into a structured Excel table, ready for analysis or regulatory use.


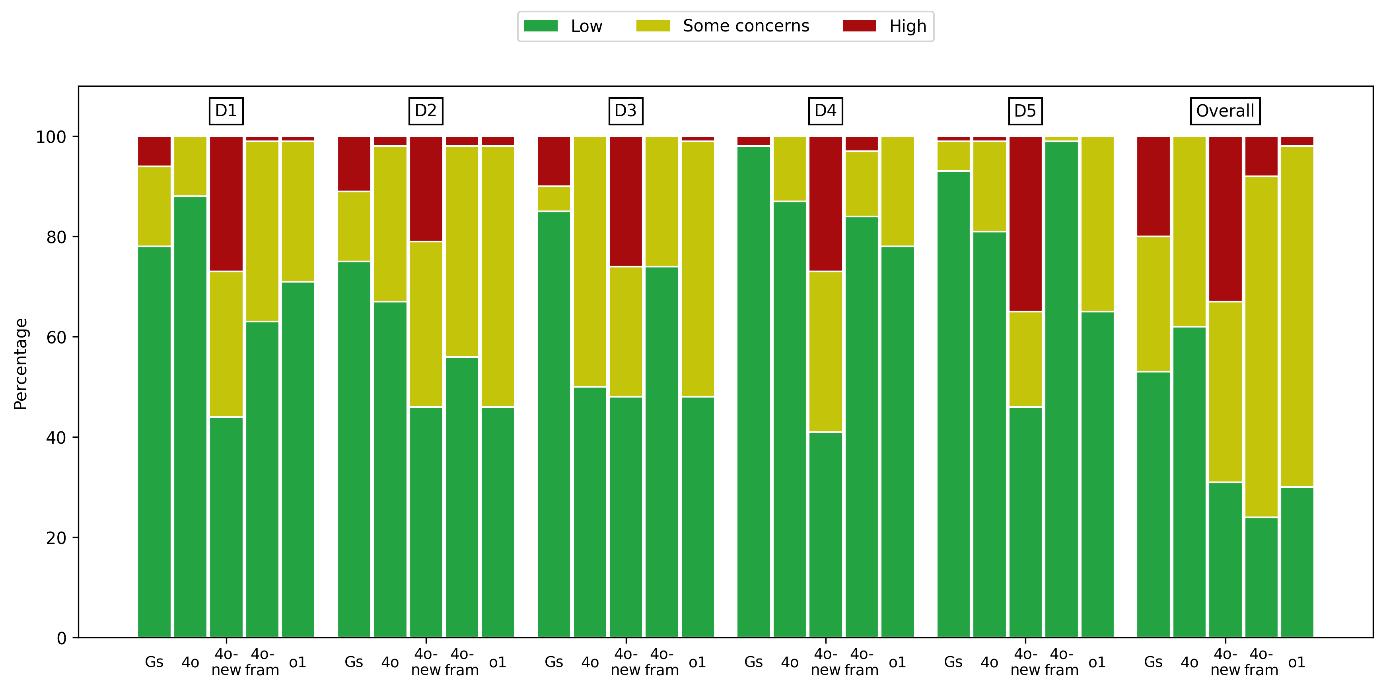


Supplementary Figure 2 Comparison of the categorization of risk of bias assessments in the five domains and overall between Gs: extracted assessments; 4o: ChatGPT-4o from Kuitunen et al.; 4o-new: ChatGPT-4o from Descamps et al.; 4o-fram: ChatGPT-4o with DeepDocs framework; o1: ChatGPT-o1; D1: Bias arising from the randomization process; D2: Bias due to deviations from the intended interventions; D3: Bias due to missing outcome data; D4: Bias in the measurement of the outcome; D5: Bias in selection of the reported results.

Supplementary Table 1 Proportion of agreement across domains and raters

| **Domain** | **4o** | **4o-new** | **4o-fram** | **o1** |
| --- | --- | --- | --- | --- |
| D1 | 80% | 42% | 73% | 65% |
| D2 | 58% | 38% | 58% | 45% |
| D3 | 50% | 41% | 68% | 46% |
| D4 | 85% | 39% | 82% | 76% |
| D5 | 74% | 43% | 92% | 62% |
| Overall | 55% | 30% | 40% | 31% |
| Global | 67% | 38.83% | 68.83% | 54.17% |
